# Contrast dependency and prior expectations in human speed perception

**DOI:** 10.1016/j.visres.2014.01.012

**Published:** 2014-02-03

**Authors:** Grigorios Sotiropoulos, Aaron R. Seitz, Peggy Seriès

**Affiliations:** a Institute for Adaptive and Neural Computation, University of Edinburgh, UK; b Department of Psychology, University of California, Riverside, USA

**Keywords:** Motion perception, Contrast, Speed prior, Bayesian models, Visual psychophysics

## Abstract

The perceived speed of moving objects has long been known to depend on image contrast. Lowering the contrast of first-order motion stimuli typically decreases perceived speed – the well-known “Thompson effect”. It has been suggested that contrast-dependent biases are the result of optimal inference by the visual system, whereby unreliable sensory information is combined with prior beliefs. The Thompson effect is thought to result from the prior belief that objects move slowly (in Bayesian terminology, a “slow speed prior”). However, there is some evidence that the Thompson effect is attenuated or even reversed at higher speeds. Does the effect of contrast on perceived speed depend on absolute speed and what does this imply for Bayesian models with a slow speed prior? We asked subjects to compare the speeds of simultaneously presented drifting gratings of different contrasts. At low contrasts (3–15%), we found that the Thompson effect was attenuated at high speeds: at 8 and 12 deg/s, perceived speed increased less with contrast than at 1 and 4 deg/s; however, at higher contrasts (15–95%), the situation was reversed. A semi-parametric Bayesian model was used to extract the subjects’ speed priors and was subsequently improved by combining it with a model of speed tuning. These novel findings regarding the dual, contrast-dependent effect of high speeds help reconcile existing conflicting literature and suggest that physiologically plausible mechanisms of representation of speed in the visual cortex may need to be incorporated into Bayesian models to account for certain subtleties of human speed perception.

## 1. Introduction

Perception has long been known to be susceptible to illusions and biases. Research on visual motion perception in particular has revealed several types of those illusions and biases, such as motion-induced blindness (Bonneh, Cooperman, & Sagi, 2001; Ramachandran et al., 1991), a preference for cardinal directions (Rauber & Treue, 1998; the motion equivalent of the *oblique effect*, Appelle, 1972), illusory “infinite regress” (Tse & Hsieh, 2006) and the dependence of perceived speed on stimulus contrast (Blakemore & Snowden, 1999; Hawken, Gegenfurtner, & Tang, 1994; Hürlimann, Kiper, & Carandini, 2002; Stocker & Simoncelli, 2006; Stone & Thompson, 1992; Thompson, 1982; Thompson, Stone, & Swash, 1996, among others). The effect of contrast on perceived speed has been extensively studied in both first (Brooks, 2001; Hawken, Gegenfurtner, & Tang, 1994; Hürlimann, Kiper, & Carandini, 2002; Stone & Thompson, 1992; Thompson, 1982) and second-order (Ledgeway & Smith, 1995) motion; in luminance-based and color-based (Cavanagh, Tyler, & Favreau, 1984; Hawken, Gegenfurtner, & Tang, 1994) motion; using narrowband (Müller & Greenlee, 1994; Thompson, 1982; Thompson, Brooks, & Hammett, 2006) and broadband (Blakemore & Snowden, 1999; Stocker & Simoncelli, 2006) artificial (such as gratings) as well as natural stimuli (such as a virtual environment simulating the viewpoint of a driver of a road vehicle Snowden, Stimpson, & Ruddle, 1998). The majority of these studies have found that decreases in contrast cause decreases in perceived speed: a high-contrast stimulus moving at the same speed as a low-contrast one appears faster.

While the effect of contrast on speed could be a limitation or an artifact of the visual system, several researchers propose that this (and other) biases may in fact be the result of optimal inference by the visual system in the face of uncertainty and internal and/or external noise. In particular, it has been suggested that perception can be thought of as unconscious inference whereby incomplete or noisy sensory information is combined with internal expectations and thus disambiguated. If these expectations match the statistics of the environment, perception is optimal, in the sense that it is the best guess about the immediate external world. This old idea (von Helmholtz, 1962) has been used to explain various phenomena in motion perception. For example, in order to explain his findings on the so-called *aperture problem*, Wallach postulated that the visual system expects objects to move slowly or be still (Wuerger, Shapley, & Rubin, 1996). The view of perception as unconscious inference has recently been formulated into the “Bayesian brain” hypothesis (Knill & Pouget, 2004; Ma, Beck, Latham, & Pouget, 2006), according to which the brain represents prior probabilities (expectations) and likelihoods (sensory input) and combines them into posterior distributions (percept) according to Bayes’ rule. In this framework, Wallach’s intuition is formalized by assuming a prior probability distribution that favors slow speeds. Such a prior has been successfully employed to explain a multitude of phenomena in motion perception (Bogadhi et al., 2011; Hedges, Stocker, & Simoncelli, 2011; Hürlimann, Kiper, & Carandini, 2002; Montagnini, Mamassian, Perrinet, Castet, & Masson, 2007; Stocker & Simoncelli, 2006; Weiss & Adelson, 1998; Weiss, Simoncelli, & Adelson, 2002), including the decrease in perceived speed as contrast decreases: at low contrasts, the sensory evidence is weak (the likelihood function is broader than at high contrasts) and therefore the influence of the prior on the final speed estimate (the mean or mode of the posterior distribution) is stronger.

However, a small number of studies have presented evidence for the opposite effect: in certain cases, low contrast results in an *increase* in perceived speed. In these studies, subjects are asked to match the speed of two drifting gratings of different contrasts presented either simultaneously or sequentially. The ratio of the actual speeds of the high- and low-contrast grating at the point of subjective equality (PSE) will be less than 1 if low contrast decreases perceived speed (the high-contrast grating will have to move more slowly in order to have the same apparent speed). Thompson (1982) found that this ratio was indeed less than 1 for temporal frequencies below 8 Hz; above that, the ratio becomes greater than 1 and contrast has the opposite effect. By conducting his experiment at a variety of spatial frequencies, he concluded that this “null point” is invariant with temporal frequency and not speed (which is the ratio of temporal to spatial frequency): regardless of the spatial frequency used, the null point was at 8 Hz. In a later study however, Stone and Thompson (1992) could not replicate this switchover at 8 Hz: in all cases, lower contrast resulted in lower perceived speed. They speculated that their earlier result was a methodological artifact (subjects making judgments other than on speed), supported by the observation that the task became very difficult at high temporal frequencies. In an attempt to settle the issue, Thompson, Brooks, and Hammett (2006) performed a similar experiment and found evidence for a null point; however, it was invariant in neither temporal frequency nor speed: at a spatial frequency of 2 cycles/deg, the null point was 6–8 Hz (i.e. at a speed of 3–4 deg/s) whereas at a spatial frequency of 8 cycles/deg, the null point was 10–14 Hz (or 1.25–1.75 deg/s). Meanwhile, data from other labs also suggested the existence of a null point at 8 Hz (Blakemore & Snowden, 1999; Hawken, Gegenfurtner, & Tang, 1994). Both studies used 1 cycle/deg gratings, corresponding to a speed of 8 deg/s. At this rate of movement, low contrast slightly increased perceived speed for all four subjected tested by Hawken, Gegenfurtner, and Tang (1994), where as Blakemore and Snowden (1999) only found this to be the case in one of the three subjects tested, although in another subject judgments were more veridical (low contrast did decrease perceived speed but less so).

Despite the wealth of data on the effect of contrast on perceived speed, the issue is not satisfactorily resolved. Is this effect a function of speed? For Bayesian models that assume a monotonically decreasing speed prior, a null point would prove problematic as it would imply a prior that increases beyond that point, at higher speeds. Can such a prior be recovered from human subjects? Furthermore, if the null point were invariant to temporal frequency and not speed, the role of a stimulus-independent speed prior for predictions would be limited.

To address these questions, we performed a speed matching experiment very similar to that by Thompson, Brooks, and Hammett (2006) using more subjects, each providing a larger number of PSE measurements than in previous studies. Experimental parameters were similar to those used in existing literature in order to allow direct comparisons. Using the Bayesian model of Stocker and Simoncelli (2006), we also extracted the priors and likelihood widths of our subjects. Furthermore, we explored the “ratio model” put forth by Thompson, Brooks, and Hammett (2006) as a non-Bayesian alternative that explains their null point findings. We found that the ratio model alone cannot account for our data but a combination of the Bayesian and ratio model offers an improvement over the Bayesian model alone.

## 2. Psychophysical experiment

### 2.1. Methods

Six subjects participated in a 5-day experiment similar to Stocker and Simoncelli (2006): a 2-AFC task where subjects were asked to compare the speeds of two drifting gratings, a reference and a test one, that were presented on a Samsung 2043BW LCD monitor on either side of a central fixation point on a uniform midgray background. Each grating was viewed through a circular aperture of 3 degrees of visual angle in diameter. The aperture centers were 6 deg to the left and right of the fixation point. The speed of the reference grating was held constant in each condition tested while the speed of the test grating was adjusted through a QUEST staircase procedure (Watson & Pelli, 1983) until the gratings appeared to move at the same speed. Each staircase terminated after 35 trials, at which point the best (the mode of the posterior pdf of the QUEST algorithm) estimate of the speed of the test grating at the point of subjective equality (PSE) was recorded. The position (left/right) of the reference grating varied randomly but was kept fixed throughout a single staircase (to minimize adaptation effects). The spatial frequency of the gratings was fixed at 2 cycles/deg – the lowest of the two values used by Thompson, Brooks, and Hammett (2006) (2 and 8 cycles/deg). Gratings had one of 3 contrast levels (3%, 15%, 95% Michelson contrast). The reference grating had one of 4 speeds (1, 4, 8 and 12 deg/s). Each condition corresponded to a unique combination of (reference and test) contrasts and reference grating speeds. Thus there were 12 conditions in total: 3 contrast level pairs (3%/15%, 3%/95% and 15%/95% for reference/test grating, respectively) times 4 reference speeds. 6 of these conditions – the ones corresponding to the lowest two speeds 1 and 4 deg/s – were grouped in a block of 420 trials in total. The remaining conditions – corresponding to the highest two speeds – were grouped in a block of 1680 trials. Thus in each of the 5 sessions, each subject performed 2 staircases for each condition in the low-speed block and 8 staircases for each condition in the high-speed block. Each trial started with a 200 ms fixation period where only the fixation point was visible, followed by a 500 ms stimulus presentation, followed by a response period in which the screen was blank (gray) until the subject pressed the left or right arrow on the keyboard to indicate which of the two gratings appeared to be moving faster. Subjects were offered an optional short break every 10 min into the experiment and a mandatory 5-min break every 20 min. The total duration of a session (day) was approximately 1 h.

The reason that high-speed trials were presented 4 times more often is twofold. First, previous research as well as pilot data indicated that speed judgments are harder at speeds above 8 deg/s and thus there is more variability in subject responses (Stocker & Simoncelli, 2006; Stone & Thompson, 1992), therefore more data is necessary to obtain an accurate estimate. Second, by presenting high speeds more often, we wished to examine whether the prior favoring slow speeds would gradually change to accommodate the stimulus statistics – i.e. moving some probability mass towards higher speeds, in a similar fashion to our previous work (Sotiropoulos, Seitz, & Seriès, 2011).

### 2.2. Results

**Fig. 2.1:**
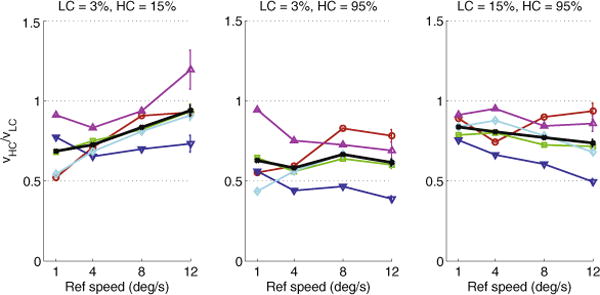
Mean ratio of speeds of the high (*ν*_*HC*_) and low-contrast (*ν*_*LC*_) gratings at the point of subjective equality (PSE), plotted as a function of speed, separately for each contrast condition. Colored points represent individual subjects; black points represent the combined data from all subjects. Error bars are standard error of the mean. (For interpretation of the references to color in this figure legend, the reader is referred to the web version of this article.)

We analyzed the PSE threshold from each staircase and report the average PSEs for each condition of each day in Fig. 2.1. Since in each trial the reference grating had a lower contrast than the test grating, we will refer to the latter as the “high contrast” grating. If lower contrast results in higher perceived speed, the ratio of speeds of the high and low-contrast grating (hereafter referred to as contrast-dependent bias – CDB) should be greater than one (because the high-contrast grating would have to move faster in order to appear as fast as the low-contrast one).

We first examined whether there was any notable effect of experience on CDB. A 3-way analysis of variance (ANOVA) on CDB with factors session number, contrast condition and reference speed showed that session had an effect on CDB (*p* < 0.04) in all but one subject. Data from two subjects that exhibited the highest effect of session is shown in Supplementary Fig. 1. However, the effect of session was nonspecific and nonmonotonic: there was no consistent change in CDB across sessions and certainly not an increase towards unity. Furthermore, when only the trials with the lowest two reference speeds are considered (where one would expect the greatest effect of exposure to the more frequent high speeds, as in Sotiropoulos, Seitz, and Seriès (2011)), ANOVAs on individual subjects’ data failed to show an effect of session (*p* > 0.1) except in one subject (where *F*_4,30_ = 3.59, *p* = 0.017). Therefore we concluded that there was no consistent perceptual change and thus data from all sessions was pooled, providing a rich data set for subsequent analysis and modeling.

We then examined whether CDB differed as a function of reference speed. Unlike in some previous reports, CDB did not exceed unity in the majority of subjects and conditions; the only exception was one subject (S5) at the lowest contrasts (3% and 15%) and highest speed (12 deg/s), although CDB was not significantly different from unity (*t*_39_ = 1.62, *p* = 0.114, two-tailed t-test). In all other cases, CDB was less than one, meaning that lowering contrast resulted in a decrease in perceived speed. In other words, apart from the aforementioned single case, there was no “null point” – a result that conflicts with that of Thompson, Brooks, and Hammett (2006).

It is worth noting the variability across subjects, especially with regards to the effect of contrast difference on perceived speed. Furthermore, when data from all subjects is pooled, there is a tendency towards more veridical perception as speed increases in the lowest-contrasts condition, seen as the positive slope of the black line in the leftmost panel of Fig. 2.1. However, the opposite tendency is seen in the highest-contrasts condition (negative slope of the black line in rightmost panel of Fig. 2.1). The aforementioned 3-way ANOVA showed that there is a marginally significant effect of speed alone (*F*_3,1440_ = 2.77, *p* = 0.0402) but a highly significant interaction effect of speed and contrast condition (*F*_6,1440_ = 9.11, *p* < 0.0001).

In summary, at contrasts below 15%, our results are in qualitative agreement with the finding of Hawken, Gegenfurtner, and Tang (1994) that as reference speed increased, the effect of contrast on perceived speed diminished and in one case even reversed. However, our results at higher contrasts (where both gratings had contrasts at least 15%) show the opposite effect: as reference speed increased, low contrast decreased perceived speed even more strongly.

## 3. Modeling

### 3.1. Methods

To model the relationship between perceived and actual speed under various contrasts and reference speeds and to extract the priors and likelihoods of our subjects, we used the Bayesian model of Stocker and Simoncelli (2006). Briefly, in each trial, an ideal observer computes estimates of the speed of each grating and chooses the grating that has a higher estimated speed. Perceived speed is assumed to be the mode of the posterior probability density function (pdf) that results from the combination of prior and likelihood. The functional form of the prior is log-linear:

(3.1) p(ν)=exp(aν+b)

*a* is the *local* slope of the logarithm of the prior: the log-prior is approximated by a straight line within a narrow speed range but the slope *a* varies with speed across larger scales (such as across points on a log scale). To reflect the dependence of the slope on speed, we will hereafter denote it by *a*(*ν*). The likelihood is Gaussian with mean equal to the true stimulus speed and width (standard deviation) separable in speed and contrast:

(3.2) σ(ν,c)=g(ν)h(c)

where the dependence on contrast, *h*(*c*), obeys a physiologically motivated inverse power law (Sclar, Maunsell, & Lennie, 1990; Stocker & Simoncelli, 2006):

(3.3) $$h\left(c\right)=\frac{1}{\sqrt{\frac{c^{q}}{c^{q}+c_{50}^{q}}r_{\max}+r_{\mathrm{base}}}}$$

The posterior distribution of the estimated speed $\hat{\nu }$ is shown to be Gaussian with mean and variance

(3.4) $$E\left(\hat{\nu }\right)=\nu +a\left(\nu \right)\sigma^{2}\left(\nu ,c\right)$$

(3.5) $$\mathrm{Var}(\hat{\nu })=\sigma^{2}(\nu ,c)$$

where *ν* is the true stimulus speed, *a*(*ν*) is the slope of the logarithm of the prior around *ν* and *σ* is the standard deviation of the likelihood function, which depends on both speed and contrast. The term *a*(*ν*)*σ*^2^(*ν*, *c*) is the prior-induced bias of the estimated speed.

The model contains 10 free parameters: 4 for each of *a* (*ν*) and *g*(*ν*) (which are not assumed to be any particular function of speed and thus require one parameter for each reference speed used in the experiment) and 2 for *h*(*c*). With 10 free parameters, optimization is not trivial and local minima cannot be avoided entirely. Stocker and Simoncelli (2006) exploit the trial-to-trial variability in the data to sufficiently constrain their model by assuming that in each trial the observer samples from the two posterior pdfs and chooses the stimulus whose sample has the highest speed value. They thus derive an expression for the psychometric function

(3.6) $$p\left({\hat{\nu }}_{2}>{\hat{\nu }}_{1}\right)=\int_{0}^{\infty }p\left({\hat{\nu }}_{2}|{\hat{\nu }}_{2}\right)\int_{0}^{{\hat{\nu }}_{2}}p\left({\hat{\nu }}_{1}|\nu_{1}\right)d{\hat{\nu }}_{1}d{\hat{\nu }}_{2}$$

where ${\hat{\nu }}_{1},\;\;{\hat{\nu }}_{2}$ are the estimated speeds of the two gratings (reference and test). Eq. (3.6) is fit to the entire dataset via a maximum-likelihood procedure.

We adopt a different, computationally cheaper, approach: given the PSE for a particular condition, the means of the posterior for each grating are equal and thus from Eq. (3.4):

(3.7) $$\nu_{1}+a\left(\nu_{1}\right)\sigma^{2}\left(\nu_{1},c_{1}\right)=\nu_{2}+a\left(\nu_{2}\right)\sigma^{2}\left(\nu_{2},c_{2}\right)$$

Since *ν* is known for both gratings, *a*(*ν*), *g*(*ν*) and *h*(*c*) can be fit to the data but because *a*(*ν*) and *σ* (*ν*, *c*) appear in a product in Eq. there are no unique best-fit values for them, i.e. the model is not sufficiently constrained as it is. However, our data consists of multiple staircases for each condition and the staircase-to-staircase variability can be exploited to constrain the model. In each session, there are 2 staircases for each of the low reference speeds and 8 for each of the high reference speeds. Since session number did not have a consistent observable effect on speed perception, data can be pooled, yielding 10 staircases for each of the low-speed conditions and 40 for each of the high-speed conditions. The squared standard error (equivalent to sample variance) $\sigma_{\mathrm{PSE}}^{2}$ of the PSE across the 10 (or 40) staircases is informative: it can be shown to be proportional to the variance of the distribution of the test speed *ν*_2_ at the PSE and inversely proportional to the number of trials in a single staircase (see Appendix A.3). The pdf of the distribution of *ν*_2_ (conditioned on the reference speed *ν*_1_ and the fact that ${\hat{\nu }}_{1}={\hat{\nu }}_{2}$ at the PSE) is Gaussian with variance equal to the sum of variances of the likelihoods of the two gratings. In particular (see Appendix A.1):

(3.8) $$p\left(\nu_{2}|\nu_{1},\;{\hat{\nu }}_{1}={\hat{\nu }}_{2}\right)\sim N\left(\nu_{1}+a\left(\nu_{1}\right)\left(\sigma_{1}^{2}-\sigma_{2}^{2}\right),\sigma_{1}^{2}-\sigma_{2}^{2}\right)$$

where *σ*(*ν*_1_, *c*_1_) is written as *σ*_1_ to reduce clutter. Thus the following equation holds:

(3.9) $$\sigma_{\mathrm{PSE}}^{2}=\alpha \frac{\sigma_{1}^{2}+\sigma_{2}^{2}}{N}$$

where *N* is the number of trials in a staircase (40 throughout our experiment) and *α* is a constant of proportionality. By comparing our fitting method against that of Stocker and Simoncelli (2006), using an independent large dataset (21 subjects) obtained with an identical stimulus and task configuration and staircase procedure (Berbec, 2013, see Appendix A.2), *α* was found approximately equal to 6.6. Eq. (3.9) thus becomes

(3.10) $$\sigma_{\mathrm{PSE}}^{2}=\frac{\sigma_{1}^{2}+\sigma_{2}^{2}}{6}$$

Using Eqs. (3.7) and (3.10), the model was fit with a least-squares procedure (lsqnonlin function, MATLAB). The (unnormalized) priors were reconstructed as in Stocker and Simoncelli (2006), by numerical integration of the fitted local slope values, according to the following equation (see Appendix A.4 for a derivation):

(3.11) p(ν)=exp(∫a(ν)dν)

where *a*(*ν*) (the slope as a function of speed) was linearly interpolated using the slope values at the 4 reference speeds. The maximum and baseline firing rates (*r*_*max*_ and *r*_*base*_) in Eq. (3.3) were set to 1 and 0.2, respectively.

### 3.2. Results

**Fig. 3.1:**
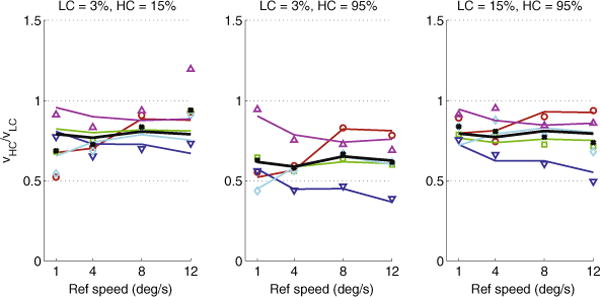
Mean ratio of speeds of the high (*ν*_*HC*_) and low-contrast (*ν*_*LC*_) gratings at the point of subjective equality (PSE), plotted as a function of speed, separately for each contrast condition. Points represent experimental data (as in Fig. 2.1); lines represent predictions of the fitted Bayesian model. Color represents individual subjects; black represents the combined data from all subjects. (For interpretation of the references to color in this figure legend, the reader is referred to the web version of this article.)

The Bayesian model fits the data reasonably well; however, as seen in Fig. 3.1, the model is unable to capture the differential effect of speed on CDB (the interaction between contrast condition and speed described in Section 2.2). This is most evident with subject S5 (magenta triangles), who shows the strongest interaction effect: the model fit is satisfactory in the last two contrast conditions (middle and rightmost panels of Fig. 3.1) but not in the first contrast condition (leftmost panel of Fig. 3.1).

**Fig. 3.2:**
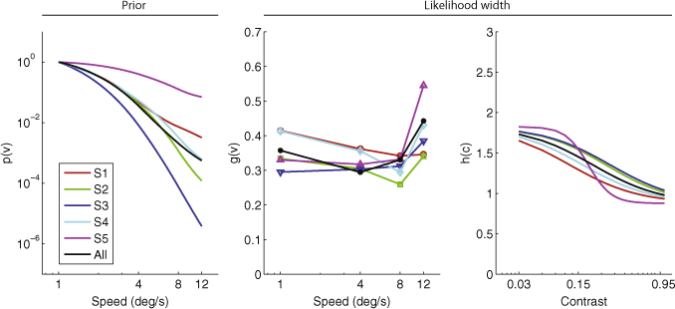
Extracted prior (left panel); speed-dependent *g*(*ν*) (middle panel) and contrast-dependent *h*(*c*) (right panel) components of likelihood width *σ*(*ν*, *c*) in the Bayesian model. Colored curves are individual subjects; black curves are all subjects combined. (For interpretation of the references to color in this figure legend, the reader is referred to the web version of this article.)

The extracted priors and likelihood widths (Fig. 3.2) are quantitatively similar to Stocker and Simoncelli (2006). The biggest difference is in the values of *g* (*ν*), which are somewhat lower for all our subjects, compared to the two representative subjects shown in Fig. 4 of Stocker and Simoncelli (2006); however, *g*(*ν*) and *h*(*c*) always appear in a product (Eq. (3.2)) and therefore there is no unique set of values for either of these functions – in other words, there is some degeneracy in the likelihood model. Discrepancies between our extracted components and those of Stocker and Simoncelli (2006) may also be due to the small differences in the stimuli (mainly the different trial duration and spatial bandwidth of the gratings) between our experiment and that of Stocker and Simoncelli (2006). It is also interesting to note the differences in extracted priors among subjects. In particular, S5 exhibits a much shallower prior than S3. Finally, as in Stocker and Simoncelli (2006), the priors for some subjects (S1 and S5) tend to flatten at the lowest and highest speeds.

The Bayesian model of Stocker and Simoncelli (2006) provides a reasonable fit to the data (*R*^2^ = 0.78, *SSE* = 0.354) but fails to account for the observed interaction effect: in the model, the effect of speed on the ratio ${\hat{\nu }}_{HC}/{\hat{\nu }}_{LC}$ is qualitatively the same across all contrasts conditions. For example, if the ratio increases with speed in one contrast condition, then it has to also increase in the other contrast conditions. This is because the same prior is used across all contrasts. Clearly, the model needs to be modified to account for the interaction. One approach is the use of a different speed prior depending on contrast level: a prior for high-contrast stimuli that has a smaller slope at low speeds than the prior for low-contrast stimuli (and vice versa at high speeds). However, there is no good theoretical or empirical justification for such a non-parsimonious approach and its many necessary assumptions.

Another approach is to model the interaction at the level of the speed measurement, which corresponds to the likelihood mean in the Bayesian model. In particular, an interaction effect would be possible if the average value of the speed measurements depended on the physical stimulus speed and contrast in a nonlinear fashion, such as through a product. One possible choice of such a nonlinearity would be the modification of Eq. (3.4) to

(3.12) $$E\left(\hat{\nu }\right)=f\left(\nu ,c\right)+a\left(\nu \right)\sigma^{2}\left(\nu ,c\right)$$

where *f* (*ν*, *c*) is no longer the true stimulus speed but a nonlinear function of true speed and contrast. Such a nonlinearity has been proposed by opponents of Bayesian models of speed perception in an attempt to explain the speed-dependent effect of contrast on perceived speed (Thompson, Brooks, & Hammett, 2006). In their “ratio model”, itself an extension of the Weighted Intersection Model (WIM) of Perrone and Thiele (2002), perceived speed is given by the ratio of a low-pass and a band-pass temporal filter. Since these filters were originally proposed to model speed tuning as a result of motion-sensitive neurons in V1, it is natural to apply them at an earlier stage than the Bayesian computations (thought to be carried out in area MT, Stocker & Simoncelli, 2006). Such an early stage naturally corresponds to modifying the actual stimulus speed used as input to the Bayesian model – that is, modifying the mean of the likelihood.

The two filters proposed by Thompson, Brooks, and Hammett (2006), low-pass and band-pass, are inseparable functions of temporal frequency (*ω*) and contrast (*c*) and their responses are given, respectively, by:

$$\begin{array}{l} p\left(\omega ,c\right)=\frac{\bar{p}\left(\omega \right)c}{\bar{p}\left(\omega \right)c+S_{p}}\\ m\left(\omega ,c\right)=\frac{\bar{m}\left(\omega \right)c}{\bar{m}\left(\omega \right)c+S_{m}} \end{array}$$

with

$$\begin{array}{l} \bar{p}\left(\omega \right)=\sqrt{a^{2}+b^{2}}\\ \bar{m}\left(\omega \right)=\frac{\omega }{k}\bar{p}\left(\omega \right)\\ a={\left({\left(2\pi \omega \tau_{1}\right)}^{2}+1\right)}^{-9/2}\\ b={\left({\left(2\pi \omega \tau_{2}\right)}^{2}+1\right)}^{-10/2} \end{array}$$

*τ*_1_ and *τ*_2_ are time constants, and *s*_*p*_ and *s*_*m*_ are semi-saturation constants of the filters. Perceived speed, as a function of temporal frequency and contrast, is then given by

(3.13) $$\nu \left(\omega ,c\right)=\frac{m\left(\omega ,c\right)}{p\left(\omega ,c\right)}$$

Eq. (3.13) thus provides the nonlinearity *f* (*ν*, *c*) used in Eq. (3.12).

**Fig. 3.3:**
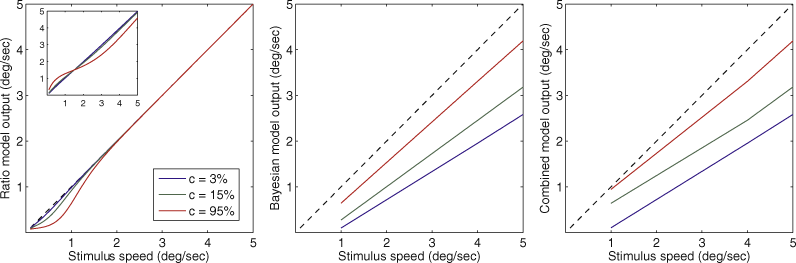
Output of the ratio model as a function of stimulus speed for the 3 contrast values used in the experiment (left panel). Parameter values are from the combined (ratio + Bayesian) model that best fits the entire data. Inset shows the ratio model output with the parameter values used by Thompson, Brooks, and Hammett (2006); “perceived speed” of the best-fit Bayesian (middle panel) and combined (right panel) models as a function of stimulus speed. Diagonal dashed line corresponds to veridical perception.

To avoid adding new free parameters to the model, we used a nested optimization procedure to find the best-fitting values for these parameters and fixed them across all subjects and conditions before fitting the parameters of the Bayesian model. We found that the best-fitting value for both *s*_*p*_ and *s*_*m*_ is 0.5, which is within the range of values used in (Thompson, Brooks, & Hammett, 2006). The other 3 parameters, *τ*_1_, *τ*_2_ and *k*, which in Thompson, Brooks, and Hammett (2006) (who followed Perrone & Thiele (2002)) were fixed to 0.0072, 0.0043 (both in units of seconds) and 4 (dimensionless), respectively, had to be changed for out data. In particular, *k* was set to 0.55 and the time constants was scaled by 4.9, yielding 0.0353 and 0.0211 for *τ*_1_ and *τ*_2_, respectively. With these parameter values, the output of the ratio model is equal to the true stimulus speed at all contrasts, except at low speeds (up to 2 deg/s), where speed mildly *decreases* with contrast (Fig. 3.3, left panel).

**Fig. 3.4:**
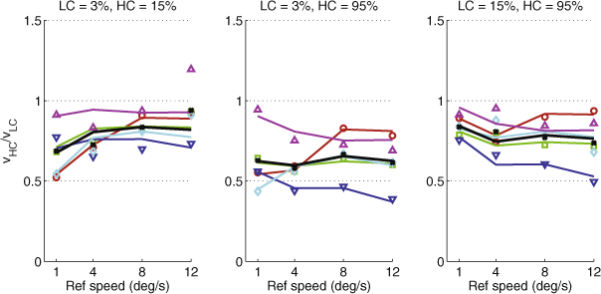
Mean ratio of speeds of the high (*ν*_*HC*_) and low-contrast (*ν*_*LC*_) gratings at the point of subjective equality (PSE), plotted as a function of speed, separately for each contrast condition. Points represent experimental data (as in Fig. 2.1); lines represent predictions of the fitted combined (ratio + Bayesian) model. Color represents individual subjects; black represents the combined data from all subjects. (For interpretation of the references to color in this figure legend, the reader is referred to the web version of this article.)

By incorporating the ratio model of Thompson, Brooks, and Hammett (2006) in the Bayesian model of Stocker and Simoncelli (2006), we were able to provide a better description of our data (Fig. 3.4), partially accounting for the interaction effect of speed and contrast and yielding a 31% improvement in the fits (*R*^2^ = 0.85, *SSE* = 0.243). This is remarkable given that the number of free parameters in the combined model is the same as in the Bayesian model (namely 10). Note that treating the rest of the ratio model parameters as free resulted in minimal further improvement in fits – too small to justify the increased model complexity.

It should also be noted that the ratio model on its own is not able to account for our data, even if all of its parameters are free. The main reason for this is that there is no set of parameter values that results in a increase in perceived speed with increasing contrast, as is found in our data: up to a certain (low) stimulus speed, which corresponds to the null point reported by Thompson, Brooks, and Hammett (2006), the output of the ratio model (corresponding to perceived speed) is an increasing function of contrast but beyond that speed the model output is a decreasing or constant function of contrast, across the entire parameter space. In the Bayesian and combined models, however, perceived speed increases with contrast across all stimulus speeds, as seen in our data (Fig. 3.3).

The opposite effects that the ratio model and the prior of the Bayesian model have on perceived speed at low stimulus speeds (around 1 deg/s) are responsible for the improved performance of the combined model. At high contrasts, the prior-induced decrease in perceived speed is attenuated at low speeds due to the ratio model, matching the data better (Fig. 3.4, right panel). This attenuation could not be provided solely by the prior because it would have to apply to all contrast conditions and thus would not fit the data well.

**Fig. 3.5:**
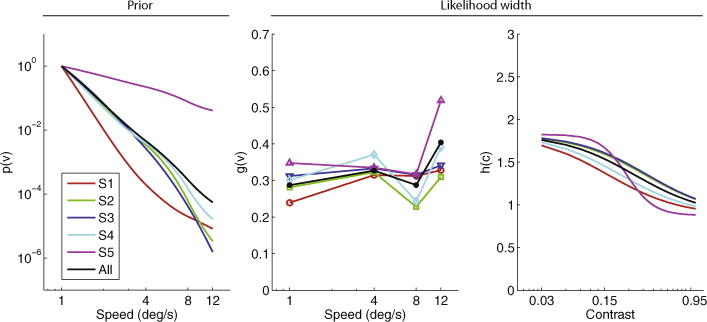
Extracted prior (left panel); speed-dependent *g*(*ν*) (middle panel) and contrast-dependent *h*(*c*) (right panel) components of likelihood width *σ*(*ν*; *c*) in the combined (ratio + Bayesian) model. Colored curves are individual subjects; black curves are all subjects combined. (For interpretation of the references to color in this figure legend, the reader is referred to the web version of this article.)

The extracted priors under the combined model are quantitatively similar to those of the original Bayesian model, with the exception of one subject (S1), whose prior is significantly steeper under the combined model (Fig. 3.5).

## 4. Discussion

Qualitatively, our results replicate the majority of existing literature in finding that lower contrast decreases perceived speed in all conditions tested. Only in one of the five subjects, at the highest speed (12 deg/s) and only at the lowest contrasts (3% and 15% Michelson contrast for the two gratings) tested was there an inversion of this relationship, although this did not reach significance.

When we quantitatively examine the data, however, we see that at the lowest tested contrasts, CDB decreases as speed increases (Fig. 2.1, leftmost panel): lowering contrast does not decrease perceived speed as much when speeds are high. Interestingly, the situation is reversed at the highest tested contrasts (15% and 95% Michelson contrast) and CDB becomes more prominent at high speeds.

Does, then, this differential effect of contrast at high speeds depend on absolute contrast levels? Data from existing literature are mixed. Among the studies that have shown evidence that decreasing contrast increases perceived speed (Blakemore & Snowden, 1999; Hawken, Gegenfurtner, & Tang, 1994; Thompson, 1982; Thompson, Brooks, & Hammett, 2006), only that of Hawken, Gegenfurtner, and Tang (1994) used contrasts as low as the lowest ones used in our study. In the other 3 studies, a reference grating of either 25% (Thompson, 1982), 64% (Blakemore & Snowden, 1999) or 70% (Thompson, Brooks, & Hammett, 2006) was matched against test gratings of lower contrasts. Since these three studies contradict our findings at comparable levels of contrast, a natural question is: are there systematic differences in experimental parameters (other than speed and contrast) between these 3 studies and the rest of the literature (including the present study) that shows evidence of a decrease in perceived speed with decreasing contrast?.

We suggest that temporal frequency is not such a parameter; we used temporal frequencies at least as high as those used by all studies that found an increase in perceived speed with decreased contrast (Blakemore & Snowden, 1999; Hawken, Gegenfurtner, & Tang, 1994; Thompson, 1982; Thompson, Brooks, & Hammett, 2006). If there were a “null point” in the temporal frequency axis, our experiment ought to have hit it. Spatial frequency is likely not a factor either – we used the same value as in one of the conditions in Thompson, Brooks, and Hammett (2006) (2 cycles/deg). The same holds for other stimulus parameters, such as the type, location and drift direction of the gratings – all these parameters were similar in conflicting studies.

Procedural differences are also unlikely to explain why we failed to find the null point. One possible factor could be the different methods of determining the PSE. However, most studies utilized staircase procedures, often very similar – e.g. Blakemore and Snowden (1999) used the same maximum-likelihood-based procedure (Watson & Pelli, 1983) that we did. Another possible factor is the task design: the two gratings could be presented simultaneously or successively and there are reports that such manipulations are important (Blakemore & Snowden, 1999; Stone & Thompson, 1992); indeed, two of the studies that conflict with ours used successive presentations (Blakemore & Snowden, 1999; Thompson, Brooks, & Hammett, 2006). However, the other two conflicting studies used simultaneous presentations (Hawken, Gegenfurtner, & Tang, 1994; Thompson, 1982).

It is possible that biases and strategies not directly related to speed perception have an effect, which may also be interactive with the experimental design: certain biases/strategies may be employed only on certain experimental setups. For example, when subjects are highly uncertain about the relative speed of two gratings, they may be inclined to pick the grating of the higher contrast as being the faster one (because it is also the most salient). Similar biases have been observed in 2-AFC experiments of orientation discrimination (Eero Simoncelli, personal communication). Furthermore, differences in the way subjects are instructed to perform the task may also play a role. For example, we have seen in our lab that subjects sometimes differ in the strategies and response biases they might use when dealing with uncertainty, even if the experimental conditions are identical, and in some cases this was due to subtle differences in instructions. There could also be a “threshold” effect of stimulus uncertainty: at very low contrasts and high speeds (high uncertainty), subjects may switch to a semi-random response strategy, for example by alternating “left” and “right” keypresses.

In regards to modeling, we have presented in this work a significantly faster model fitting procedure than that used by Stocker and Simoncelli (2006); instead of using every trial of every staircase per condition, we used just the final estimate of the staircase plus the variability of this estimate across staircases. Effectively, we fit the model using just 1=*N*th of the data, where *N* is the number of trials in a single staircase (40 in our case). Using an independent large dataset (Berbec, 2013, see Appendix A.2), we compared our fitting method to that of Stocker and Simoncelli (2006) and, like them, we used the likelihood of the data under the best-fitting model as a performance metric, whereby 100% corresponds to the likelihood of the data when separate Weibull functions for each condition are fit to it and 0% the likelihood under the random (coin-flipping) model. Over the entire dataset, the performance of our fitting method is 87%, compared to 93% of the method of Stocker and Simoncelli (2006). The extracted prior and likelihood components were also very similar between the two methods. Therefore our method can be useful during model selection/design, allowing rapid iteration between fitting and design, until a suitable model is found, which can then be fit with the method of Stocker and Simoncelli (2006) for slightly more accurate quantitative predictions. Our method could also be used in cases where not every trial of the staircase is available, such as when modeling data from existing literature (where usually only the PSE is reported).

By incorporating the ratio model of Thompson, Brooks, and Hammett (2006) (which in itself is unable to describe our data, also see Fig. 3.3) as a pre-processing step in the Bayesian model of Stocker and Simoncelli (2006), we were able to provide an improved account of the interaction effect of contrast and speed. However, this improvement is restricted to low speeds (around 1 deg/s), where an increase in contrast causes a mild decrease in speed; there is no improvement at high speeds (around 12 deg/s). The observed interaction could be better accounted for by a model in which perceived speed (prior to the Bayesian computations) decreases with contrast at low speeds but increases with contrast at high speeds. The simple ratio model of Thompson, Brooks, and Hammett (2006) can only produce the former effect – it cannot produce an increase in perceived speed with contrast at high speeds. It would be interesting to examine whether an extended version of the ratio model, for example one that incorporates band-pass filters tuned to various temporal frequencies, could better account for the interaction; this is left as future work. We also note that, unlike the Bayesian model, the ratio model seemingly constitutes a departure from a normative explanation of speed perception. However, the ratio model was proposed, in the form of the WIM model (Perrone & Thiele, 2002; Perrone, 2005), as a biologically plausible way of achieving variable speed tuning in MT neurons by using a small number of V1 neurons tuned not to speeds but to a *limited* range of spatial and temporal frequencies. Thus, while the WIM model does not result in optimal perception, it can be be argued that this is due to biological constraints earlier in the visual hierarchy (V1) rather than an inherent suboptimality in the model, in much the same way as the Bayesian model is optimal under the assumption of noisy earlier measurements.

## Supplementary Material

Sup

## Appendix A. Supplementary material

Supplementary data associated with this article can be found, in the online version, at http://dx.doi.org/10.1016/j.visres.2014.01.012.
