# Supplementary material for: Contrast dependency and prior expectations in human speed perception
Source: Vision Res. Author manuscript; Available in PMC 2016 Jun 21. (PMC4915944; doi:10.1016/j.visres.2014.01.012)
Supplement: Sup [file NIHMS793266-supplement-Sup.pdf]

Appendix A. Supplementary Procedures

Appendix A.1. Derivation of Equation 3.8

The perceived speeds of the two gratings, $\hat{v}_1$ and $\hat{v}_2$ are normally distributed random variables (Equations 3.4 and 3.5) and thus can be written as

$$\begin{aligned}\hat{v}_1 &= v_1 + a(v_1)\sigma_1^2 + \sigma_1^2 s_1 \\ \hat{v}_2 &= v_2 + a(v_2)\sigma_2^2 + \sigma_2^2 s_2\end{aligned}$$

where s_1 and s_2 are independent standard Gaussian random variables ($\sim \mathcal{N}(0,1)$). At the PSE, $\hat{v}_1 = \hat{v}_2$ i.e. $v_2 = v_1 + a(v_1)\sigma_1^2 - a(v_2)\sigma_2^2 + \sigma_1^2 s_1 - \sigma_2^2 s_2$. Thus v_2 is a (weighted) sum of independent normal random variables plus a constant and therefore it is itself a normal random variable with mean $v_1 + a(v_1)\sigma_1^2 - a(v_2)\sigma_2^2$ and variance $\sigma_1^2 + \sigma_2^2$. Since the logarithm of the prior is assumed locally linear in the model, $a(v_1) = a(v_2)$ and Equation 3.8 follows directly.

Appendix A.2. Model validation dataset

Following up to the present study, additional data was collected by Dan Berbec as part of his Honours project under the supervision of Peggy Seriès (Berbec, 2013), with the view to looking at different aspects of the topic of speed priors. This dataset consists of a larger number of subjects (21) than our dataset; however it includes significantly fewer sessions (at most 6 staircases for each reference speed and contrast condition) per subject, particularly in the conditions with the highest two reference speeds (which show the greatest variability and thus require more data for an accurate estimate of CDB). Although some experimental parameters, such as reference speeds, differ from our experiment and thus this dataset cannot be pooled with that of the present study, it was useful for validating our method of fitting the Bayesian model of Stocker & Simoncelli (2006).

Appendix A.3. PSE Variability

Here we derive a lower bound on the variance of the test speed v_2 at the PSE. We first note that the psychometric function (Equation 3.6) presented in Stocker & Simoncelli (2006) can be simplified if we slightly rearrange the lhs as $p(\hat{v}_2 - \hat{v}_1 > 0)$. Since $\hat{v}_1$ and $\hat{v}_2$ are Gaussian variables, their difference is also Gaussian with mean $\mu_2 - \mu_1 = v_2 + a(v_2)\sigma_2^2 - v_1 - a(v_1)\sigma_1^2$ and variance $\sigma_1^2 + \sigma_2^2$. Therefore $p(\hat{v}_2 > \hat{v}_1) = p(\hat{v}_2 - \hat{v}_1 > 0) = p(\Delta\hat{v} > 0)$ is the complement of a cumulative normal distribution

$$p(\Delta\hat{v} > 0) = \int_0^\infty p(\Delta\hat{v})d\Delta\hat{v} = \Phi(\frac{\mu_2 - \mu_1 - \Delta\hat{v}}{\sqrt{\sigma_1^2 + \sigma_2^2}}) \tag{A.1}$$

Therefore the Bayesian observer model is a type of probit analysis and thus we can use standard theoretical (Finney, 1971) and simulation (McKee et al., 1985)

results from probit analysis. The simplest analytical formula for the standard error (SE) of the staircase threshold estimate (in our case T_{50}) is

$$\sigma_{PSE} = \frac{\sigma}{\sqrt{\sum wn}} \tag{A.2}$$

where $\sigma = \sqrt{\sigma_1^2 + \sigma_2^2}$ is the slope of the psychometric function, n is the number of trials at each stimulus level and w are the probit weights. As McKee et al. (1985) point out, this is a variation of the common statistical formula $s/\sqrt{N}$ used to estimate the standard error of the mean. The similarity between the two formulas makes intuitive sense if we think of a staircase procedure as an estimator of the mean of the underlying distribution of the test speed v_2 at the PSE (Equation 3.8). In their simulations, McKee et al. (1985) found that the SE of a staircase estimate for T_{75} (the threshold at 75%) in a 2-AFC paradigm is approximately $3\sigma/\sqrt{N}$ (in the variable-slope case) and also that the SE of T_{50} in a yes-no paradigm is approximately half of that. Furthermore, they assert that under certain conditions of trial placement, these are lower bounds for any possible staircase procedure. Since our staircase procedure was designed to estimate T_{50} and $p(\hat{v}_2 > \hat{v}_1)$ corresponds to a yes-no paradigm, it follows that

$$\sigma_{PSE} \geq \frac{3\sigma}{2\sqrt{N}} = \frac{3\sqrt{\sigma_1^2 + \sigma_2^2}}{2\sqrt{40}}$$

and thus

$$\sigma_{PSE}^2 \geq \frac{\sigma_1^2 + \sigma_2^2}{17.7} \tag{A.3}$$

This lower bound is tighter when the number of trials per staircase is relatively small, typically less than 60 (McKee et al., 1985), such as in our experiment. Our empirically determined SE, whose square is given by Equation 3.10, is in broad agreement with these results.

Appendix A.4. Derivation of Equation 3.11

The logarithm of the prior is assumed a locally linear function of speed (see Equation 3.1), that is

$$f(v) = \ln p(v) = a(v)v + b$$

Thus, within a small range of speeds Δv , the following holds:

$$f(v + \Delta v) = f(v) + a(v)\Delta v$$

or

$$a(v) = \frac{f(v + \Delta v) - f(v)}{\Delta v} \tag{A.4}$$

Letting $\Delta v \to 0$, the right-hand side of Equation A.4 is simply the derivative of $f(v)$, i.e. $a(v) = \frac{df(v)}{dv}$. Integrating both sides, this becomes

$$f(v) = \int a(v)dv + C$$

and thus

$$p(v) = exp(f(v)) = exp(\int a(v)dv + C) = C'exp(\int a(v)dv)$$

where $C' = exp(C)$ plays the role of a normalizing constant, i.e. its value is such that $\int_0^\infty p(v)dv = 1$.

Appendix B. Supplementary Figures

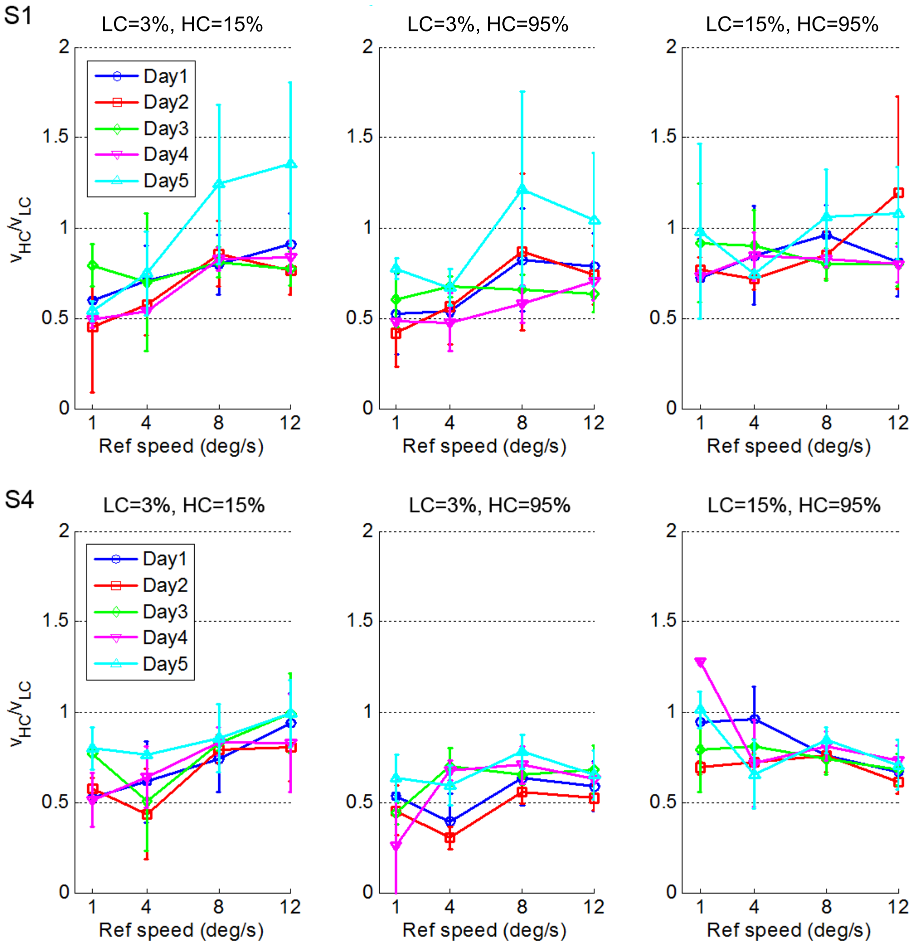

Figure B.1: Mean ratio of speeds of the high (v_{HC}) and low-contrast (v_{LC}) gratings at the point of subjective equality (PSE), plotted as a function of speed, separately for each experimental session and contrast condition. Data from two subjects (S1 and S4) who showed the greatest inter-session variability. Error bars are std.dev.
